# Nitrogen Metabolism Disorder Accelerates Occurrence and Development of Lung Adenocarcinoma: A Bioinformatic Analysis and *In Vitro* Experiments

**DOI:** 10.3389/fonc.2022.916777

**Published:** 2022-07-12

**Authors:** Zexin Zhang, Zhikai Xiahou, Wenfeng Wu, Yafeng Song

**Affiliations:** ^1^ The First Clinical Medical College, Guangzhou University of Chinese Medicine, Guangzhou, China; ^2^ China Institute of Sport and Health Science, Beijing Sport University, Beijing, China; ^3^ The Second Clinical Medical College, Guangzhou University of Chinese Medicine, Guangzhou, China

**Keywords:** nitrogen metabolism disorder, lung adenocarcinoma, immune regulation, prognostic model, nomogram

## Abstract

**Background:**

Nitrogen metabolism (NM) plays a pivotal role in immune regulation and the occurrence and development of cancers. The aim of this study was to construct a prognostic model and nomogram using NM-related genes for the evaluation of patients with lung adenocarcinoma (LUAD).

**Methods:**

The differentially expressed genes (DEGs) related to NM were acquired from The Cancer Genome Atlas (TCGA) database. Consistent clustering analysis was used to divide them into different modules, and differentially expressed genes and survival analysis were performed. The survival information of patients was combined with the expressing levels of NM-related genes that extracted from TCGA and Gene Expression Omnibus (GEO) databases. Subsequently, univariate Cox analysis and the least absolute shrinkage and selection operator (LASSO) regression were used to build a prognostic model. GO and KEGG analysis were elaborated in relation with the mechanisms of NM disorder (NMD). Meanwhile, immune cells and immune functions related to NMD were discussed. A nomogram was built according to the univariate and multivariate Cox analysis to identify independent risk factors. Finally, real-time fluorescent quantitative PCR (RT-PCR) and Western bolt (WB) were used to verify the expression level of hub genes.

**Results:**

There were 138 differential NM-related genes that were divided into two gene modules. Sixteen NM-related genes were used to build a prognostic model and the receiver operating characteristic curve (ROC) showed that the efficiency was reliable. GO and KEGG analysis suggested that NMD accelerated development of LUAD through the Wnt signaling pathway. The level of activated dendritic cells (aDCs) and type II interferon response in the low-risk group was higher than that of the high-risk group. A nomogram was constructed based on ABCC2, HMGA2, and TN stages, which was identified as four independent risk factors. Finally, RT-PCR and WB showed that CDH17, IGF2BP1, IGFBP1, ABCC2, and HMGA2 were differently expressed between human lung fibroblast (HLF) cells and cancer cells.

**Conclusions:**

High NM levels were revealed as a poor prognosis of LUAD. NMD regulates immune system through affecting aDCs and type II interferon response. The prognostic model with NM-related genes could be used to effectively evaluate the outcomes of patients.

## Background

Nitrogen plays a pivotal role in biological processes as the most abundant element in the air and wildly exists in natural world. It is common knowledge that nitrogen is an essential element for protein composition, RNA and DNA (1). Generally, nitrogen is derived from proteolysis in foods (2). Nitrogen can also come from proteolysis of muscle. Therefore, the level of nitrogen in human body is generally used to assessing nutritional status (3, 4). In many cases, decreased level of nitrogen is found in most cancer patients due to the consumption of tumor cell (5).

In human body, nitrogen was mainly excreted in skin, feces, and urine after producing. The urea cycle is the major pathway to metabolic nitrogen (6). After synthesis of toxic substance ammonia from nitrogen, ammonia is transformed to low toxic urea through urea cycle in liver and excreted *via* kidney and urine (7). In many cancers, inactivation of special enzymes in urea cycle has shown to increase the utilization of nitrogen to the synthesis of pyrimidine and support the synthesis of RNA and DNA of cancer cell, thus accelerating the development of cancers (8). Therefore, the study of the correlations between utilization of nitrogen and cancers is warranted.

The increasing demand of nitrogen has been recognized as one of the six hallmarks of metabolism of cancers (9). Even if the consumption of carbon will be increased in the synthesis of biological processes, the demand of nitrogen is decreased accompanying growth signals (10). As a matter of fact, many nitrogenous molecules such as nucleotide, nonessential amino acids, and polyamine must be synthesized in proliferating cells *de novo* (5). A non-essential amino acid containing two reduced nitrogen atoms, glutamine, is the main transport pathway to reduce nitrogen in metazoan cells (11).

In addition, it has been shown that the tumor micro-environment (TME) is in a state of hypoxia (12). Glutamine is utilized as the major carbon source for the biological synthesis in hypoxic TME (13). However, the excessive utilization of carbon in glutamine indirectly causes remnant of nitrogen. Subsequently, the remnant of nitrogen will be used to synthesize nucleotide of cells. Nevertheless, it is harmful for cells if nitrogen is transformed to the synthesis of ammonia in human body (14). Therefore, avoiding damage of ammonia to cancer cell is important. On the one hand, studies have confirmed that MCF breast cancer cells were able to recycle and metabolize ammonia that releasing from the nitrogen of glutamine (14, 15). On the other hand, generally, it is supposed that glutamine can release the amide nitrogen to synthesize ammonia and convert it to glutamate. Furthermore, it is able to generate α-ketoglutarate and expel the accumulated ammonia through deamination or transamination (16). Therefore, study of how cancer cells utilize nitrogen is meaningful.

However, the relationships between nitrogen metabolism disorder (NMD) and lung adenocarcinoma (LUAD) is not fully understood and genes that play a significant role in NM are not identified. Additionally, the relationship between NMD and immune function also remains unknown. In this research, we developed a model of prognosis for the evaluation of patients with LUAD and used external data cohorts to verify its efficiency through bioinformatic analysis. At last, *in vitro* experiments were used to verify these predictive results.

## Methods and Materials

### Identification of Differentially Expressed Nitrogen Metabolism-Related Genes

NM-related genes were acquired from Gene Set Enrichment Analysis (GSEA) database (https://www.gsea-msigdb.org/gsea/index.jsp) by setting the key word as “Nitrogen metabolism” and species as “Homo sapiens” with the criterion of Min Enrichment as 1.5, Min overlap as 3, and *p* value cutoff as 0. 01 (**Supplementary Table 1**). Afterward, the expressed data and metadata profile of LUAD were downloaded from TCGA database (https://portal.gdc.cancer.gov/). The microarray data of GSE42127 and its corresponding platform file GPL6884 with 176 tumor samples, and microarray data of GSE68571 and its corresponding platform file GPL80 with 86 tumor samples were downloaded from GEO database (https://www.ncbi.nlm.nih.gov/geo/). The limma package in R software was used to screen the NM-related genes with DEGs between tumor and normal tissues. *p* value <0.05 was considered to be significantly different.

### Clustering Analysis on NM-Related Gene Modules

ConsensusClusterPlus was used to carry out cluster analysis of NM-related genes, which was using agglomerative pam clustering with a 1-Pearson correlation distance and 80% of the samples were resampling for 10 repetitions. The optimal number of clusters was determined using the empirical cumulative distribution function plot. Thereafter, KM survival analysis was performed to distinct existing significant difference between different gene modules. Meanwhile, clinical factors such as age, gender, and clinical stages were discussed in different gene modules. Finally, based on the criteria of log∣FC∣>1 and adjust *p* value <0.05, we used R language limma package to conduct differentially expressed analysis to identify DEGs.

### Prognostic Model Construction and Validation Based on Univariate Cox and Multi Cox Analysis

Survival package in R software was used to carry out univariate Cox analysis to filter these genes related to LUAD patients’ prognosis and LASSO regression was used to remove redundant factors to build a predictive model. Furthermore, in order to validate the reliability of this model, external data sets GSE42127 and GSE68571 were used for analysis. To further verify whether high- and low-risk scores had an impact on patients’ survival, according to the median risk scores, the patients were divided into two groups and then tested using KM survival analysis. Univariate Cox analysis and multivariate Cox analysis were also used to test whether the risk scores of the model was an independent prognostic factor. In addition, principal component analysis (PCA) and T-distributed Stochastic Neighborhood Embedding (tNSE) analysis were used for data quality control.

### Mechanistic Analysis of Risk Scores in the Occurrence and Development of LUAD

To further understand whether the risk scores of NM disorder affected clinical features, limma and heatmap package were used to analyze whether there were existing significant difference among different groups. Additionally, we also performed differentially expressed gene analysis to screen out DEGs with the criteria of log∣FC∣>1 and adjust *p* value <0.05 between the high- and low-risk groups. Subsequently, we carried out GO and KEGG functional analysis for the DEGs using the online Metascape database (http://metascape.org/gp/index.html#/main/step1) to explore the mechanisms of risk scores of LUAD.

### Differentially Expressed Gene Analysis on Risk Scores in the Immune Cells and Immune Functions

To further understand whether the risk scores of NM disorder affected immune cells and immune functions, we used GSVA package, GSEABase package, and limma package for further analysis. Immune cells included: activated dendritic cells (aDCs), B cells, CD8+ T cells, dendritic cells (DCs), immature dendritic cells (iDCs), macrophages, mast cells, neutrophils, natural killer cells (NK cells), plasmacytoid dendritic cells (pDCs), T helper cells, T follicular helper cells (Tfh), T-helper 1 cells (Th1 cells), T-helper 2 cells (Th2 cells), tumor infiltrating lymphocyte (TIL), and regulatory T cells (Treg) were evaluated. Immune functions included: antigen-presenting cell co-inhibition (APC co_inhibition), antigen-presenting cell co-stimulation (APC_co_stimulation), C-C chemokine receptor (CCR), check-point, Cytolytic_activity, human lymphocyte antigen (HLA), inflammation-promoting, major histocompatibility complex class I (MHC_class_I), parainflammation, T_cell_co-inhibition, T_cell_co-stimulation, type I interferon response (Type_I_IFN_Reponse), and type II interferon response (Type_II_IFN_Reponse) were also evaluated.

### Construction of a Nomogram for Nitrogen Metabolism Disorder and Lung Adenocarcinoma

Prognostic related genes of NM in the predictive model and clinical manifestations were incorporated into Cox risk analysis. The independent prognostic risk factors were identified and used to construct a nomogram. Subsequently, a forest plot was draw by R package “forestplot”. A nomogram was developed based on the results of multivariate Cox analysis to predict the overall survival of patients among different years. All of the elements were given a representing legend and were used to calculate the risk scores in the nomogram. By summing the scores of all elements, we got a total scores and it is corresponding survival time. Then, we used C-index to evaluate the reliability of the model and correction curve to evaluate high and low of the fitting degree. If the calibration curve of nomogram model is more closer to the fitting curve, the ability of its prediction is more better.

### Hub Nitrogen Metabolism Related-Gene Screening

In order to screen the hub genes related to NM in lung adenocarcinoma, the PPI protein network was constructed for the prognostic genes screened by univariate Cox analysis. Specifically, we import the genes into the String database (https://cn.string-db.org/cgi/input.pl?sessionId=jjziGhJsy4od&amp; Input_page_active_form =multiple_identifiers), which was the output into the protein interaction network. Furthermore, the Degree calculation method of cytoHubba plug-in in Cytoscape3.7.2 identify the 5 hub genes. Then, the candidate hub genes were obtained by intersection processing of 5 core genes and prognostic model genes. Finally, the expression levels of mRNA and protein of these genes were examined by RT-PCR and WB in human lung fibroblast (HLF) cells and three kinds of LUAD cells.

### Cell Culture

HLF (PCS-201-013™), human lung adenocarcinoma A549 (CCL-185™), human lung cancer NCI-H460 (HTB-177™) cells, and human non-small cell lung cancer NCI-H1299 cells (CRL-5803) were obtained from ATCC. HLF cells were cultured in Fibroblast Basal Medium (ATCC, PCS-201-030). NCI-H460 and NCI-H1299 cells were cultured in RPMI 1640 medium (Gibco, C11875500BT). A549 cell was cultured in F12-K medium (ATCC, 30-2204). All media were supplemented with 10% heat-inactivated fetal bovine serum (FBS; Gibco, 10099-141), 1% penicillin–streptomycin (Gibco, 15070063), and 2 mM glutamine (Gibco, 15140-122). The cells were kept in a 37°C incubator with 5% CO_2_. All cell lines were detected for mycoplasma contamination by PCR.

### RNA Extraction and Real-Time Fluorescence Quantitative PCR

Total RNA was extracted from the above four cell lines using Trizol reagent (*In vivo*Gen, 15596018). The cell culture medium was aspirated, an appropriate amount of Trizol was added, transferred to a sterilized 1.5 mL centrifuge tube, and stood at room temperature for 5 min. Then, 1/5 Trizol volume of chloroform was added, shaken violently, and let stand for 5 min at room temperature. Subsequently, centrifuge was refrigerated at 4°C and centrifuged at 12,000×*g* for 15 min to transfer the supernatant to the new EP tubes and isopropanol solution with the same volume as supernatant was added, mixed gently, and let stand at room temperature for 15 min. Centrifuge was set as 4°C and 12,000×*g* for 15min. The supernatant was discarded and 1 mL 75% ethanol was added into the EP tube to wash the precipitation. Refrigerated centrifuge was set as 4°C, centrifuged 12,000×*g* for 10 min, and supernatant was discarded. Then the pellets were dried and added DEPC H_2_O to dissolve. The following reaction solution was prepared in a microcentrifuge tube to synthesize cDNA by reverse transcription with a total volume of 20 μL (**Table 1**). Afterward, the cDNA was placed at room temperature for 10 min, then transferred to 42°C for 1 h, and cooled in ice water for 2 min. The detail of the RT-PCR reaction was shown in **Table 2**. Finally, CFX96 real-time quantitative fluorescence PCR system (Bio-RAD) was used for real-time quantitative polymerase chain reaction analysis. GAPDH was used as a normal internal control, and the relative expression levels of genes were calculated and analyzed by 2^-ΔΔCt^ method. Each sample was measured three times. Primer sequences were shown in **Table 3**.

**Table 1:** cDNA.

Template RNA solution	1ug
5×Reverse Transcriptase Buffer	4ul
dNTP Mixture(10 mM each)	2ul
RNase Inhibitor	20U
Oligo (dT) 18 Primer	50 pmol
AMV Reverse Transcriptase	10 U
DEPC H2O	up to 20ul

**Table 2:** RT-PCR reaction system.

SYBR Green I (2×)	10ul
primer F (10uM)	1ul
primer R (10uM)	1ul
cDNA	0.2ul
add ddH2O to	20ul

**Table 3:** Primer sequences.

Primer name	Primer sequence (5′-3′)
HMGA2-homo-qF	GCCCCAGGAAGCAGCAGCAA
HMGA2-homo-qR	AGGTCTGCCTCTTGGCCGTT
RHOV-homo-qF	CTTGAGTGCTCAGCCTTGAC
RHOV-homo-qR	GCACACCTTTGGCATTCAGT
IGFBP1-homo-qF	AAGGCTCTCCATGTCACCAA
IGFBP1-homo-qR	TGTCTCACACTGTCTGCTGT
FBN2-homo-qF	GCTTTGTGGAGCAAAGGGAA
FBN2-homo-qR	GTAGCCACCCAGGATGTTCT
CDH17-homo-qF	TCTGCAGTTGTGTGGAAGGA
CDH17-homo-qR	ATCACCAGAAGGGTGGTCAG
ABCC2-homo-qF	CAGGTTTGCCGGCGATATTT
ABCC2-homo-qR	GGCGGGAGGTAGACACATAA
ITGB7-homo-qF	AAATCTATGACCGCCGGGAA
ITGB7-homo-qR	AAGCGAGGATTGATGGTGGT
MELTF-homo-qF	AACAGCCAGGAGCGGTATTA
MELTF-homo-qR	TGGCCGTTTGTGTTGTCAAA
GAPDH-homo-qF	GGACTCATGACCACAGTCCA
GAPDH-homo-qR	TCAGCTCAGGGATGACCTTG

### Western Blot

The above four kinds of cells were lysed with RIPA lysis buffer (Thermo, 89901) at 4°C for 30 min and centrifuged at 12,000×*g* for 30 min. The supernatant is then transferred to a new centrifuge tube placed on ice and the precipitate is discarded. Then the protein concentration was determined by BCA method, and the same amount of protein and molecular weight standard were sampled into SDS-PAGE gel well. The total protein was sampled at 20–30 μg, and the electrophoresis was performed at 100 V for 1 to 2 h. The proteins were then transferred from the gel to the PVDF membrane and sealed with a blocking solution for 1 h. Primary and secondary antibodies were added, respectively. Through the automatic detection of chemiluminescence image analysis system, the expression of ABCC2, CDH17, HMGA2, IGF2B1, IGFBP1, and GADPH was detected.

## Results

### Identification of Differentially Expressed Nitrogen Metabolism-Related Genes

**Figure 1:**
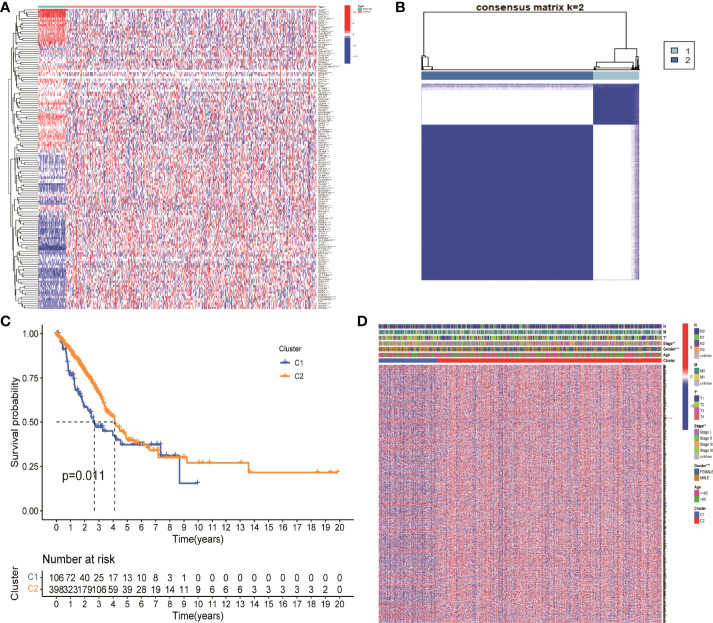
Identification of differentially expressed genes associated with nitrogen metabolism in lung adenocarcinoma. **(A)** Differential expression analysis identified 138 genes related to nitrogen metabolism in lung adenocarcinoma. **(B)** Consistent cluster analysis divided 138 genes into two gene modules. **(C)** KM survival analysis showed significant differences between C1 and C2 gene modules. **(D)** Clinical factors such as sex, clinical stage, and T stage showed significant differences between the two gene modules.

There were 203 genes related to NM which was finally obtained from GSEA database. Fifty nine normal samples and 535 tumor samples were included in TCGA LUAD. The expression of 203 genes was extracted from the expression matrix of TCGA LUAD at first. Then, differentially expressed analysis identified 138 DEGs. *p* value <0.05 was considered as a significant difference. An asterisk (*) represents *p* < 0.05; two asterisks (**) represent *p* < 0.01, and three asterisks (***) represent *p* < 0.001 (**Figure 1A**).

### Consistent Clustering Analysis for Nitrogen Metabolism-Related Gene Modules

The optimal CDF value was screened out and divided the NM genes into two gene modules by consistent clustering analysis (**Figure 1B**). KM survival analysis showed that the difference between the two modules C1 and C2 was significant. The survival status of patients in module 2 was significantly better than that in module 1, with a *p* value of 0.011 (**Figure 1C**). In addition, gender, clinical stage, and T stage were significantly different between the two modules, which suggested that these two modules can be used not only for clinical staging of patients, but also for patient prognosis assessment (**Figure 1D**). Finally, the expression levels of 694 DEGs in NM were extracted from the differential expression analysis of the two gene modules for further analysis.

### Prognostic Model Construction and Validation Based on Univariate Cox and Multivariate Cox Analysis

**Figure 2:**
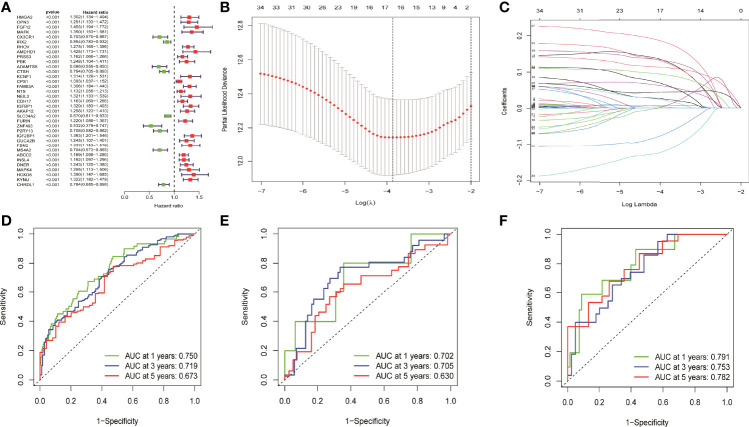
Univariate cox analysis and LASSO regression constructed and verified the prognostic model. **(A)** Thirty five prognostic genes were screened by univariate cox analysis. **(B, C)** LASSO regression removed the redundancy factors and constructed a prognostic model with 16 genes. **(D–F)** The ROC curves of TCGA LUAD, GSE42127, and GSE68571 verified the reliability of the model.

**Figure 3:**
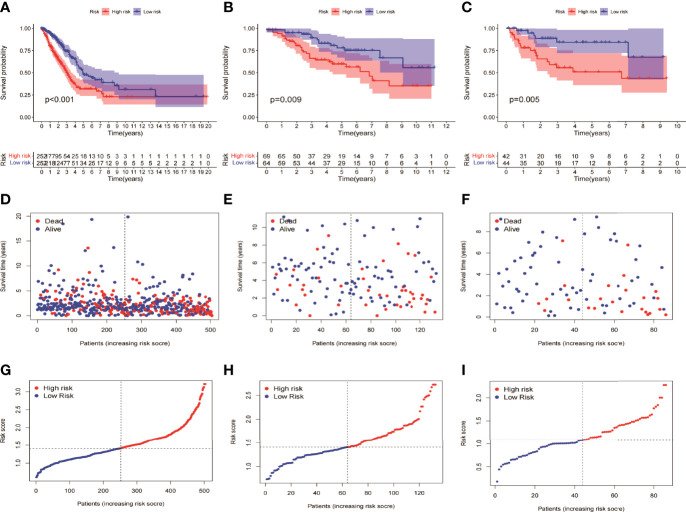
KM survival analysis between the high- and low-risk groups of the model. **(A, D, G)** TCGA LUAD showed a worse prognosis in the high-risk group than in the low-risk group, with *p* < 0.001. **(B, E, H)** GSE42127 showed that the prognosis of the high-risk group was worse than that of the low-risk group (*p* = 0.009). **(C, F, I)** GSE68571 showed that the prognosis of the high-risk group was worse than that of the low-risk group (*p* = 0.005).

**Figure 4:**
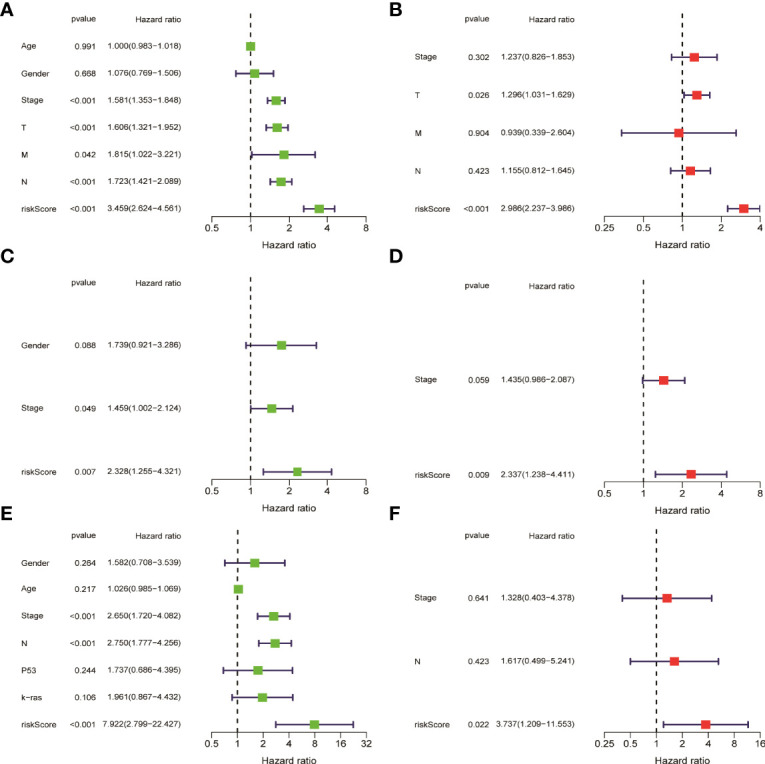
Univariate cox analysis and multivariate Cox analysis verified that the risk score of the model was an independent prognostic risk factor. **(A, B)** TCGA LUAD. **(C, D)** GSE42127. **(E, F)** GSE68571.

**Figure 5:**
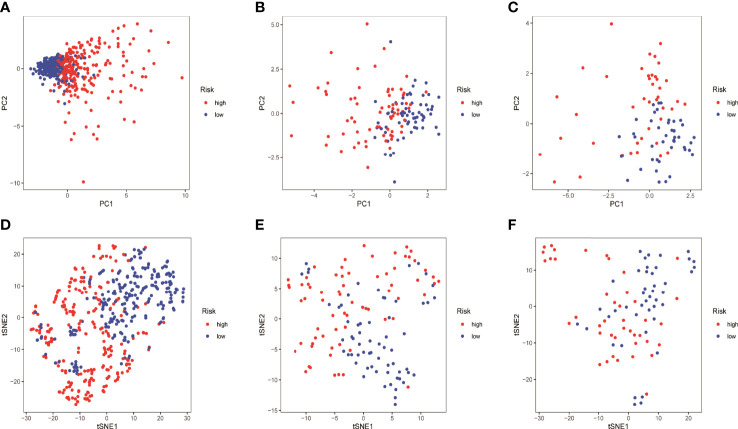
Quality of data control by PCA and tNSE analysis. **(A–D)** TCGA LUAD. **(B–E)** GSE42127. **(C–F)** GSE68571.

Totally, 35 prognostic related genes were identified based on the univariate Cox analysis of 694 DEGs in NM with the criteria of *p* < 0.001, of which most was related to the poor prognosis of LUAD patients with an HR value greater than 1 (**Figure 2A**). Then, LASSO regression was used to remove redundant factors and 16 genes were used to construct a prognostic model (**Figure 2B, C**). The ROC curve of the training set was 0.75 for 1 year, 0.719 for 3 years, and 0.673 for 5 years, indicating the reliability of the model (**Figure 2D**). Meanwhile, the ROC curve of the external data set GSE42127 were 0.702 for 1 year, 0.705 for 3 years, and 0.630 for 5 years (**Figure 2E**). The ROC curve of the external data set GSE68571 were 0.791 for 1 year, 0.753 for 3 years, and 0.782 for 5 years (**Figure 2F**). These results verified the reliability of the model. The formula of risk scores was as follow:

HMGA2*0.129+RHOV*0.044+PRSS3*0.003+KCNF1*0.025+FAM83A*0.112+CDH17*0.021+IGFBP1*0.131+ZNF493*-0.071+P2RY13*-0.117+IGF2BP1*0.006+FBN2*0.181+ABCC2*0.007+DNER*0.060+MAPK4*0.064+HOXD8*0.126+KYNU*0.092. KM survival analysis showed that there were significant differences between the high- and low-risk groups in the three data sets. The survival time of the high-risk group of NM was significantly lower than that of the low-risk group of NM (**Figures 3A–C**). With the increase of risk, the number of deaths in LUAD patients increased significantly, suggesting that LUAD patients with high NM risk have poor prognosis (**Figures 3D–I**). Further univariate Cox analysis and multivariate Cox analysis confirmed this, showing that the risk scores of the model was an independent prognostic factor in all three data sets with HR > 1 (**Figure 4**). In terms of data quality control, PCA analysis and tNSE analysis showed that NM risk scores could significantly distinguish different groups of patients, further proving the reliability of our risk score model (**Figure 5**).

### Mechanistic Analysis of Risk Scores in the Occurrence and Development of LUAD

**Figure 6:**
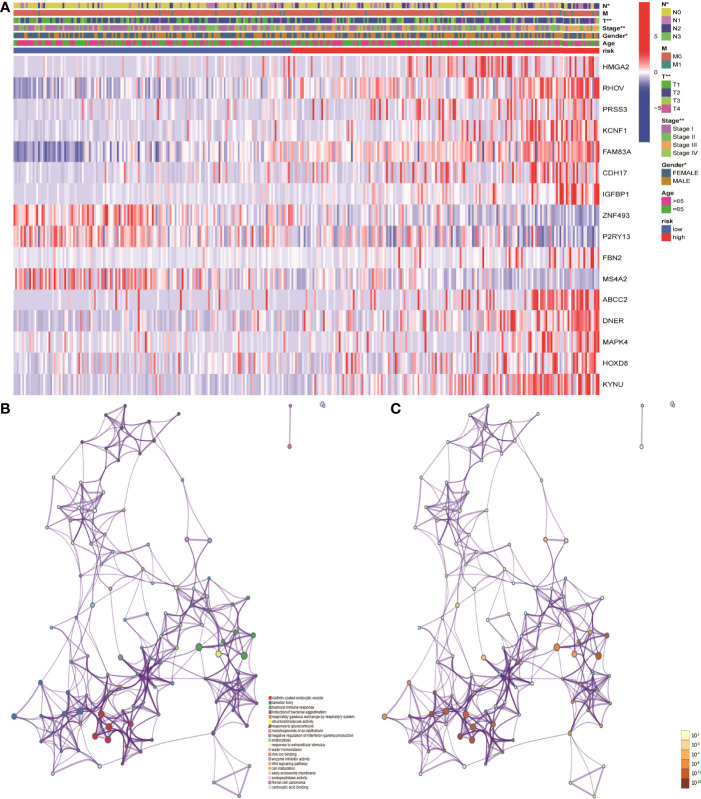
Mechanistic analysis of the involvement of risk scores in tumor occurrence and development. **(A)** Clinical manifestations such as clinical stage, gender and TN stages showed significant differences between the high- and low- risk groups. **(B, C)** GO and KEGG functional analysis showed that the risk scores in tumor development play a role through biological functions such as humoral immune response and pathways such as Wnt signaling pathway.

In order to further understand the mechanism of risk scores in the occurrence and development of LUAD, the risk scores were combined with age, gender, clinical stage, and TNM stages. The results showed that the risk scores in gender, clinical stage, and TN stages have significant differences, suggesting that risk scores can be used as test indicators for clinical grades (**Figure 6A**). Furthermore, 86 DEGs were identified by differentially expressed analysis between high- and low-risk groups. GO and KEGG functional enrichment analyses were performed using the online Metascape database. The results showed that the mechanism of risk score involved in tumor development may play a role through biological functions such as humoral immune response and Wnt signaling pathway (**Figures 6B, C**).

### Differentially Expressed Analysis on Risk Scores in the Immune Cells and Immune Functions

**Figure 7:**
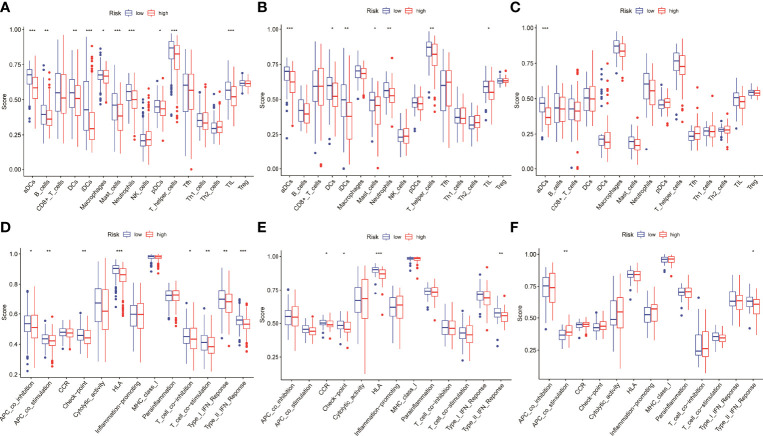
Differential expression analysis of risk scores involved in immune. **(A–C)** The aDCs was significantly higher in the low-risk group in the three datasets. **(D–F)** The type II interferon response was significantly higher in the low-risk group than in the high-risk group in the three datasets.

To further understand whether the risk scores was related to immune cells and immune functions, the three were combined and then significance level tests were performed. The results showed that aDCs in the low-risk group was significantly higher than that in the high-risk group in the three data sets (**Figures 7A–C**). Immune function analysis showed that type II interferon response of low-risk group in the three datasets of was significantly higher than the high-risk group, which may be related to the better prognosis of the patients (**Figures 7D–F**).

### Construction of a Nomogram for Nitrogen Metabolism Disorder and Lung Adenocarcinoma

**Figure 8:**
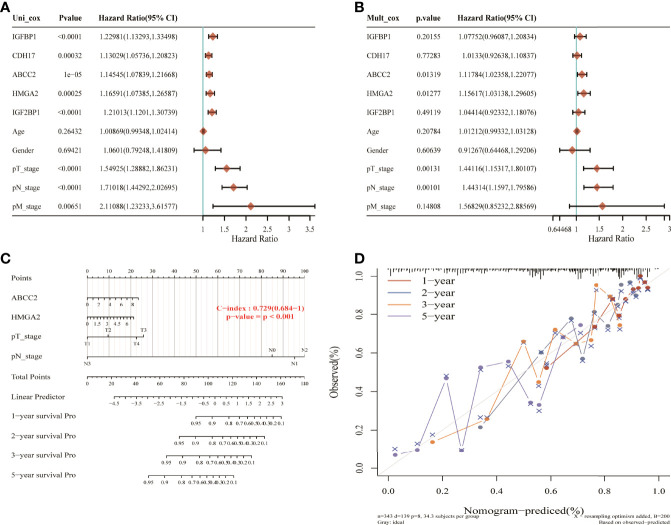
Nomogram construction of nitrogen metabolism genes in lung adenocarcinoma. **(A, B)** ABCC2, HMGA2M, T, and N stages were identified as four independent prognostic risk factors. **(C)** The nomogram constructed using ABCC2, HMGA2M, T, and N stages with C-index of 0.729 illustrated the model reliability. **(D)** The correction curves for 1, 2, 3, and 5 years showed good fit of the model.

Using univariate Cox analysis and multivariate Cox analysis, 16 NM-related genes, age, sex, and TNM stage were included in the prognostic model of LUAD. The results showed that ABCC2, HMGA2, T, and N stage were independent prognostic risk factors (**Figures 8A, B**). These four factors were constructed in the nomogram (**Figure 8C**). The results show that its C-index is 0.729 and *p* value is <0.001, which indicates the reliability of the nomogram. In addition, the correction curve is evenly distributed in the diagonal manner, indicating that the model fits well (**Figure 8D**).

### RT-PCR and Western Blot Verified the Expression of Nitrogen Metabolism Related Genes in Lung Adenocarcinoma

**Figure 9:**
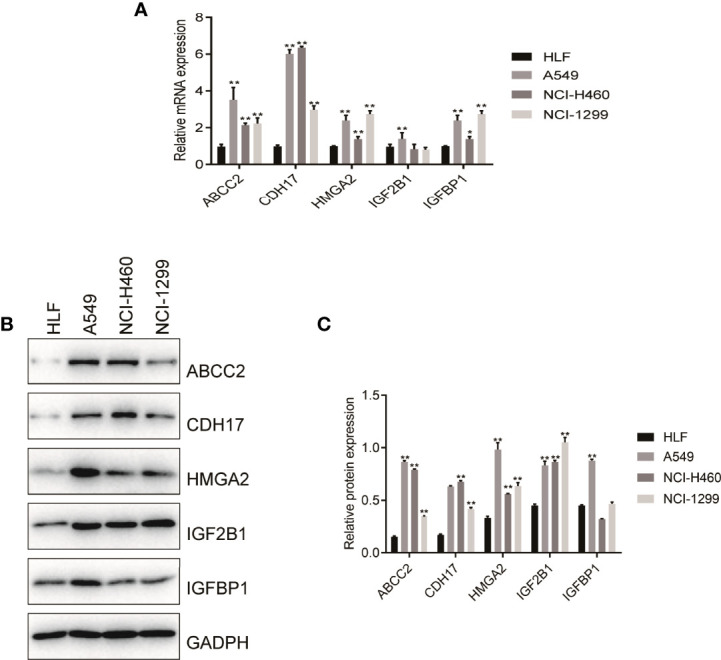
RT-PCR and Western blot verified the expression level of nitrogen metabolism related genes in lung adenocarcinoma. **(A)** RT-PCR verified the mRNA expression of NM-related genes in normal human cell and in tumor cells. **(B, C)** Western blot validated protein of NM-related genes expressing levels in normal human cell and in tumor cells.

According to the size of the Degree value of cytoHubba plug-ins, the 5 key genes including CDH17, IGF2BP1, IGFBP1, ABCC2, and HMGA2 were selected. Subsequently, by intersecting the 10 core genes with the 16 model genes, five candidate genes were identified, including CDH17, IGF2BP1, IGFBP1, ABCC2, and HMGA2. In order to verify these predictive results, the expressing levels of the 5 genes in HLF cells and tumor cells were performed by RT-PCR and WB experiments. The results showed that the mRNA levels of CDH17, IGFBP1, ABCC2, and HMGA2 were significantly overexpressed in A549, NCI-H460, and NCI-1299 cells compared with HLF cells, while IGF2BP1 was only overexpressed in A549 cells compared with HLF cells, suggesting that these 5 genes may be associated with poor prognosis of patients (**Figure 9A**). Further, WB experiment showed that ABCC2, HMGA2, and IGF2BP1 protein levels were significantly higher in A549, NCI-H460, and NCI-1299 cells compared with HLF cells, and CDH17 protein level was significantly higher in NCI-H460 and NCI-1299 cells compared with HLF cells. IGFBP1 was significantly overexpressed only in A549 compared with HLF cells, suggesting that these five genes may be associated with poor prognosis in patients (**Figures 9B, C**).

## Discussion

Nitrogen metabolism is as important as glucose metabolism because it provides nitrogen source for the synthesis of nucleotide and protein in cancer cells. As the important feature of proliferation of tumors and immune cells, NMD can affect cell metabolism and oncogenic programs (5). Therefore, to know the pathways and mechanisms of nitrogen metabolism in human body is very important. As a matter of fact, in our human body cells, nitrogen is mainly derived from amino acids and nucleotides, of which glutamine is second to glucose to serve as one of most rich amino acid in blood (17). In fact, glutamine not only provide carbon source for proliferating cells, but play a pivotal role in providing nitrogen source and supporting redox homeostasis (10). Notably, glutaminase 2 (GLS2) is overexpressed in lung carcinoma and colorectal cancer (17).

Additionally, it was reported that NMD was closely related to the TME. In TME, cancer-associated fibroblasts (CAFs) contribute in the development, occurrence, and immunosuppression of cancers as a producer of extracellular matrix and paracrine signal. It has previously been shown that ammonia can activate CAF autophagy signaling pathway thus secreting high level of glutamine to TME to support the cancer cells uptake and growth. It is well-known that autophagy and senescent cell markers are overexpressed in lung cancer stromal cells (18, 19). In addition, nitrogen of glutamine is necessary to activate T cells in TME, whereas the removal of tryptophan can suppress T cell function in TME, indicating that NMD in TME holds substantial promise (5). Unfortunately, there are few studies on NMD in TME. Therefore, the mechanisms of NMD in TME of lung adenocarcinoma (LUAD) should be evaluated comprehensively.

To elaborate the mechanisms of NMD in LUAD, 16 NM-related genes were screened out by Cox LASSO regression model and were used to develop a prognostic model for evaluation of LUAD patients. The results showed that the AUC of the training cohort was 0.750 for 1 year, 0.719 for 3 years, and 0.673 for 5 years, suggesting that the model was able to effectively evaluate the outcome of LUAD patients. This result was verified by external cohorts with the AUC 0.702 for 1 year, 0.705 for 3 years, and 0.630 for 5 years in the GSE42127 cohort. The AUC was 0.791 for 1 year, 0.753 for 3 years, and 0.782 for 5 years in the GSE68571 cohort. Finally, five hub genes HMGA2, IGFBP1, CDH17, ABCC2, and IGF2BP1 were obtained through intersection with genes in the prognostic model and PPI protein interaction network.

High mobility group protein A2 (HMGA2) is involved in important processes such as cell replication, recombination and repair of DNA, which is a non-histone structural transcription factor (20). On one hand, HMGA2 has a strong transformation regulatory capacity in malignancies (21). For example, it can promote breast cancer to escape apoptosis in coordination with Bach-1 (22). And the ectopic expression of HMGA2 was able to increase the proliferation and migration capacity of A549 cells in LUAD (20). On the other hand, HMGA2 can bind to nucleotide sites which are rich in adenine and thymine (23). It might be related to HMGA2 assisting nitrogen flow to nucleotide biosynthesis in tumor cells. Study has been confirmed that insulin-like growth factor-binding protein-1 (IGFBP-1) can mediate insulin sensitivity to provide tumor cells with the glucose needed for growth in tumor cells (24). The expression of IGFBP1 is closely related to the overall prognosis of non-small cell lung cancer (NSCLC), especially patients with lung adenocarcinoma (25). Some studies have suggested that the anabolic process of IGF-1 and IGFBP1 may make use of substrates such as amino acids to promote tumor development (26). Liver-intestine cadherin (cadherin 17, CDH17) can regulate tumor cell apoptosis signaling pathway, which is associated with the proliferation and adhesion of malignant tumors including gastric cancer, pancreatic cancer and so on (27, 28). The mechanism of action of CDH17 in lung adenocarcinoma has not been reported, but its relationship with lung intestinal adenocarcinoma and tumor lung metastasis has been confirmed (29, 30). CDH17 had been found to be characterized by single-nucleotide polymorphisms in lung adenocarcinoma in a proteomic study (31). CDH17 is knocked down affects cell cycle, signal transduction and cell proliferation (32). Overexpression of ABCC2 in A549 cells is associated with cisplatin tolerance. It means that targeting ABCC2 is able to serve as a therapeutic choice for multi-drug resistance in patients with NSCLC (33). ABCC2 and GSH can co-transport efflux drugs to reduce drug accumulation and cytotoxicity in tumor cells (34). Weak basic drugs mainly rely on ABCC2 for co-transport with GSH, while precursor amino acids are essential for GSH synthesis (35). Thus, ABCC2 may induce an increased GSH synthesis to promote tumor progression, associated with nitrogen metabolism. Insulin-like growth factor 2mRNA-binding protein-1 (IGF2BP1) can be targeted to bind to mRNA or ncRNA, which plays significant effects in the occurrence of tumors and embryo. It has shown that IGF2BP1 is overexpressed in NSCLC and significant negatively related to survival in lung adenocarcinoma in both *in vivo* and *in vitro* studies. Among them, miR-494 may regulate the levels of IGF2BP1 and IGF2mRNA through a nitrogen metabolism mechanism, which causes A549 cell senescence (36).

Additionally, ABCC2, HMGA2, and TN stages were identified as independent prognostic risk factors through univariate and multivariate Cox analysis, and they were used to construct a nomogram for LUAD. The results showed that the nomogram achieved good performance for the evaluation of survival status of LUAD patients with a C-index of 0.729 (0.684-1), *p* < 0.001.

Our study found that the involvement of NM in the occurrence and development of LUAD may be related to humoral immune response and played a role through Wnt signaling pathway. Wnt signaling plays a key role in embryonic development and maintenance of adult tissue homeostasis (37). The dysfunction of Wnt signaling pathway is associated with the progression of many cancers, and promotes immunosuppression in TME by regulating the differentiation and maturation of CD4+ Tregs and other variety of inflammatory cells (38). In addition, Wnt pathway can regulate decomposition of glutamine and synthesis of nucleotide in tumor tissues by targeting MYC to participate in NM, causing NMD (39). In addition, Twist1 activates the Wnt signaling pathway and upregulates the activity of β-catenin/TCF complex, which enhances gene transcription of cancer cells and activates downstream target genes to influence EMT and participate in cell adhesion process, thus accelerating the progression of lung cancer (40, 41). We found that the Wnt signaling pathway was correlated with the identified genes to varying degrees. For example, in many cancers such as colorectal cancer (42) and endometrial cancer (43), HMGA2 and Wnt signaling pathway have a synergistic regulatory effect. Increased classical Wnt signaling and cell proliferation were observed in the lungs of HMGA2 knockout mice (44). HOXA13-targeted regulation of CDH17 can affect the Wnt/β-catenin signaling pathway, thus, affecting the progression of gastric cancer (45).

Furthermore, we found that the level of aDCs and response to Type II IFN in the low-risk group was higher than in the high-risk group, suggesting that it was related to the better prognosis of LUAD patients. DCs are important antigen presenting cells, and reduction of DCs in peripheral blood of patients with non-small cell lung cancer is related to tumor development (46). One study showed that CD73-ADC treatment can induce a strong intracellular accumulation of pro-inflammatory macrophages and activated dendritic cells in lung cancer cells, which is effective in inhibiting CD73-dysregulated tumors and improving immune responses (47). DCs can present antigens to T and B cells and sensitize CD4+ and CD8+ lymphocytes to secrete cytokines including interferon γ to inhibit and destroy tumor tissues (46). Similar to type I and III interferon, IFN-γ plays an anti-tumor immune role by reducing cell proliferation, enhancing the number and types of antigen clusters, and promoting the phagocytosis of macrophages (48). Increased IFN-γ secretion of CD8+ cells was found in mouse lung cancer models, which downregulated the anti-tumor effect of PD-1 by promoting the activation of mature DC cells (49). It has been suggested that classical Wnt signaling pathway can regulate DC function and play an indispensable role in shaping anti-tumor immunity. Immature or tolerant DCs exhibit catabolism such as fatty acid oxidation and increased glutamine decomposition, while immunogenic or inflammatory DCs exhibit glycolysis as a marker of anabolism. More importantly, the Wnt signaling pathway can transform DC metabolism from anabolism to catabolism, mediating DC metabolic reprogramming in TME (50). CDC1 helps activating T cells and drives INF-γ secretion of CD8+T (51). Studies have found that the Wnt signaling pathway can eliminate the gradient generation of chemokines required by DC (52).

In order to verify the reliability of the results of bioinformatic analysis, we used RT-PCR and WB to test the expressed levels of HMGA2, IGFBP1, CDH17, ABCC2, and IGF2BP1 in HLF cell and three kinds of LUAD cells. The results showed that the mRNA levels of CDH17, IGFBP1, ABCC2, and HMGA2 were significantly overexpressed in A549, NCI-H460, and NCI-1299 cells compared with HLF cell, while IGF2BP1 was only overexpressed in A549 cells compared with HLF cell, suggesting that these 5 genes may be associated with poor prognosis of patients. Furthermore, WB experiment showed that the protein levels of ABCC2, HMGA2, and IGF2BP1 were significantly higher in A549, NCI-H460, and NCI-1299 cells compared with HLF cells, and CDH17 protein levels were significantly higher in NCI-H460 and NCI-1299 cells compared with HLF cells. IGFBP1 was significantly overexpressed only in A549 compared with HLF cells, suggesting that these five genes are associated with poor prognosis of patients.

## Conclusion

Taken together, we developed a NM-related prognostic model and nomogram for evaluating the survival status of LUAD patients. We found that 5 NM-related genes are related to the poor prognosis of LUAD. This study provides directions to study NMD and LUAD. Additionally, we found that NMD involved in the occurrence and development of LUAD was closely associated with aDCs and response to Type II IFN.

## Data Availability Statement

The original contributions presented in the study are included in the article/**Supplementary Material**. Further inquiries can be directed to the corresponding author.

## Author Contributions

ZZ: conceptualization, methodology, software, data curation, and writing original draft. ZX: conceptualization, methodology, and experiments. WW: software and data curation. YS: writing—review and editing. All authors read and approved the final manuscript.

## Funding

This study was supported by the National Natural Science Foundation of China (Grant no. 82071413).

## Conflict of Interest

The authors declare that the research was conducted in the absence of any commercial or financial relationships that could be construed as a potential conflict of interest.

## Publisher’s Note

All claims expressed in this article are solely those of the authors and do not necessarily represent those of their affiliated organizations, or those of the publisher, the editors and the reviewers. Any product that may be evaluated in this article, or claim that may be made by its manufacturer, is not guaranteed or endorsed by the publisher.

## Abbreviations

NM, nitrogen metabolism; LUAD, lung adenocarcinoma; DEGs, differentially expressed genes; TCGA, The Cancer Genome Atlas; GEO, Gene Expression Omnibus; LASSO, least absolute shrinkage and selection operator; RT-PCR, real-time polymerase chain reaction; WB, Western blot; ROC, receiver operating characteristic curve; GO, Gene Ontology; KEGG, Kyoto Encyclopedia of Genes and Genomes; GSEA, Gene Set Enrichment Analysis; KM, Kaplan–Meier; AUC, area under the curve; PCA, principal component analysis; tNSE, T-distributed Stochastic Neighborhood Embedding; aDC, activation dendritic cells; DCs, dendritic cells; iDCs, immature dendritic cells; NK cells, natural killer cells; pDCs, plasmacytoid dendritic cells; Tfh, T follicular helper cells; Th1 cells, T-helper 1 cells; Th2 cells, T-helper 2 cells; TIL, tumor infiltrating lymphocyte; Treg, regulatory T cells; APC co_inhibition, antigen-presenting cell co-inhibition; APC_co_stimulation, antigen-presenting cell co-stimulation; CCR, C-C chemokine receptor; Check-point; Cytolytic_activity; HLA, human lymphocyte antigen; MHC_class_I, major histocompatibility complex class I; Type_I_IFN_Reponse, type I interferon response; Type_II_IFN_Reponse, type II interferon response.
